# Direct evidence on Ta-Metal Phases Igniting Resistive Switching in TaO_x_ Thin Film

**DOI:** 10.1038/srep14053

**Published:** 2015-09-14

**Authors:** Min Kyu Yang, Hyunsu Ju, Gun Hwan Kim, Jeon-Kook Lee, Han-Cheol Ryu

**Affiliations:** 1 Future Convergence Research Division, Interface Control Research Center, Korea Institute Science &Technology (KIST), Seoul 136-791, Republic of Korea; 2 Department of Materials Science and Engineering, Seoul National University, Seoul 151-744, Korea; 3 Department of Car Mechatronics, Sahmyook Univ., Seoul 139-800, Republic of Korea

## Abstract

A Ta/TaO_x_/Pt stacked capacitor-like device for resistive switching was fabricated and examined. The tested device demonstrated stable resistive switching characteristics including uniform distribution of resistive switching operational parameters, highly promising endurance, and retention properties. To reveal the resistive switching mechanism of the device, micro structure analysis using high-resolution transmission electron microscope (HR-TEM) was performed. From the observation results, two different phases of Ta-metal clusters of cubic α-Ta and tetragonal β-Ta were founded in the amorphous TaO_x_ mother-matrix after the device was switched from high resistance state (HRS) to low resistance state (LRS) by externally applied voltage bias. The observed Ta metal clusters unveiled the origin of the electric conduction paths in the TaO_x_ thin film at the LRS.

Resistive switching (RS) phenomena in various transition metal oxides (TMO) have been considerably investigated for next-generation non-volatile memory application as conventional sub-micron device fabrication skill confronts its physical limitation^1^,^2^,^3^,^4^,^5^. Early stage of researches mostly focused on examining various material candidates for the RS itself. Afterward, the RS reliability issue for commercial device application led to mainly investigating a few candidate materials such as TaO_x_^6^,^7^, HfO_x_^8^,^9^, and TiO_x_^10^,^11^,^12^,^13^. Among these, TaO_x_ has been highlighted for its excellent endurance performance and low power consumption in the RS operation^6^,^7^. *Yang. et al.* reported the highly promising endurance characteristic over ~10^10^ RS cycles of the TaO_x_ thin film, which is attributed to simple thermodynamic equilibrium phases of Ta-O system in the range of conventional RS operation condition^7^. According to their report, the high endurance characteristic of the TaO_x_ thin film can benefit by two thermodynamic equilibrium phases-insulating oxide mother-matrix and electrically conductive phase. These two stable phases can accommodate electrical conduction paths and oxygen reservoir alternatively during the RS. Later, the conductive phase was identified as amorphous Ta-rich Ta(O) surrounded by nano-crystalline Ta_2_O_5_ phase^14^,^15^. However, direct observation on the Ta-rich phase as the electrical conduction path in the TaO_x_ thin film is not yet presented. In this work, the highly promising RS performance of the TaO_x_ thin film is demonstrated and microscopic analyses by high-resolution transmission electron microscope (HR-TEM) provide direct observation on the origin of the RS in the TaO_x_ thin film.

## Results

**Figure 1:**
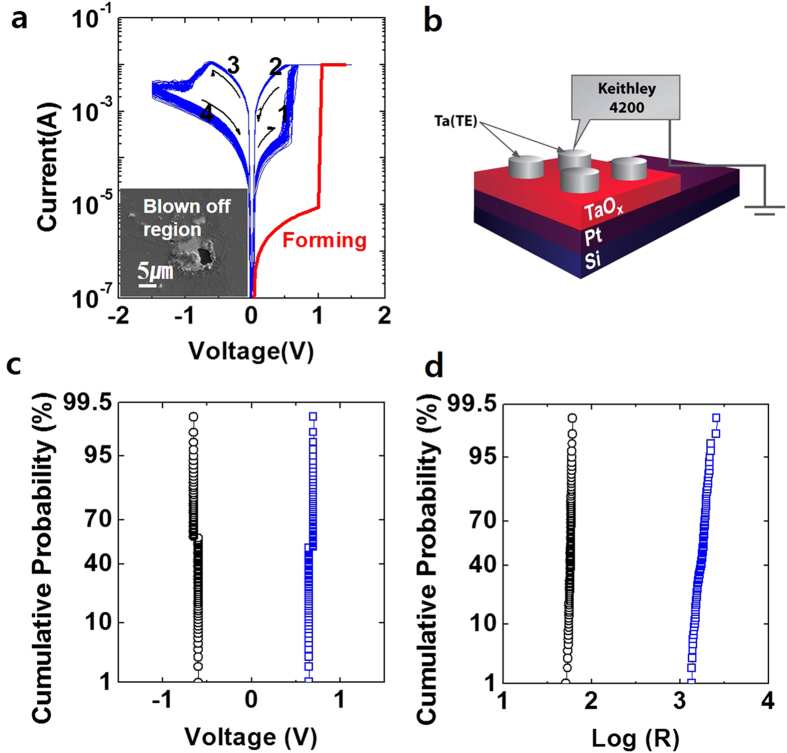
(**a**) The *I–V* characteristic of Ta/TaO_x_/Pt device and the inset presenting the blown-off region during SET operation. (**b**) The schematic figure of Ta/TaO_x_/Pt device and measurement system (**c**) and (**d**) show cumulative probability of the operation voltage and LRS/HRS resistance distribution, respectively.

Figure 1(a) shows *I–V* hysteresis characteristic of a Ta/TaO_x_/Pt capacitor-like device. The electrical measurement was performed with applying voltage bias on top electrode (Ta) and maintaining electrical ground on bottom electrode (Pt), as schematically shown in Fig. 1(b). To avoid permanent dielectric break-down, compliance current (CC) was set to 10 mA during ‘SET’ (resistance change from high resistance state (HRS) to low resistance state (LRS) by external bias) measurement. Pristine state of the device went through the initial RS cycle remarked by the red line in Fig. 1(a), so called ‘forming’ process. Over 300 RS cycles, the tested device demonstrated highly uniform distribution of SET operation voltage at ~0.65 V and of RESET (resistance change from LRS to HRS) voltage at ~–0.6 V as denoted in Fig. 1(c). Standard deviations of the SET and the RESET voltages are respectively 0.025 V and 0.024 V and the ratio of the deviation to each operation voltage is less than 4%. The resistance distributions of each LRS and HRS are presented in Fig. 1(d). It is assumed that the resistance in each state should be statistically distributed obeying a normal distribution model. Based on this assumption, the peak-to-peak separation between the LRS and the HRS was estimated by difference between the mean resistance of the LRS and the HRS and was 6.27 (σ_HRS_ + σ_LRS_), where the standard deviations of the HRS and the LRS resistance are σ_HRS_ and σ_LRS_, respectively. This assures that the probability of the mutual overlap between the resistance states is less than approximately 0.0002 ppm, which can be interpreted that less than one out of 5 Giga bits may be identified as an error. In addition to the promising RS *I–V* characteristics of the device, severely damaged region in the top Ta electrode was microscopically observed after the RS measurement as shown in the inset of Fig. 1(a). The similar observation on TiO_x_ thin films has already been reported by others^12^,^16^. It is attributed to sudden evolution of oxygen gas within the thin films during the RS to induce the rupture of the top electrode. This blown off region provides a useful site to observe the origin of the RS with unveiling the causality of the rupture. The detailed observation results will be discussed in the following.

**Figure 2:**
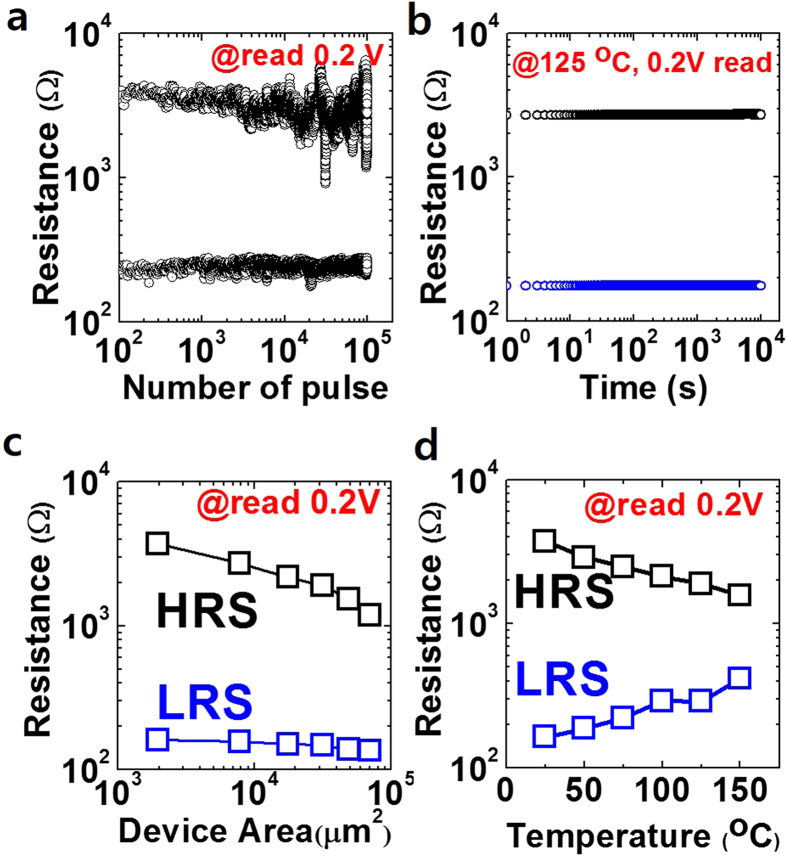
(**a**) and (**b**) show the electrical endurance and the retention characteristics of Ta/TaO_x_/Pt device, respectively. (**c**) and (**d**) exhibits the device size and temperature dependent behaviour of LRS and HRS, respectively.

The reliability characteristics of the tested device were examined and the electrical endurance and the retention characteristic are shown in Fig. 2 (a), respectively. The current level of the device was read at reading voltage of 0.2 V for the endurance and the retention measurements and the tested device was kept at 125 °C for accelerated condition of the retention measurement. The device demonstrated the reliable electrical endurance of up to 10^5 ^RS cycles and the retention characteristic of 10^4 ^sec. The retention characteristic was also certified in various temperature conditions, data not shown here, and the reliable retention characteristic had been confirmed. Although the electrical endurance performance of the tested device in this study was lower than one in the previous report by *Yang et al.* 7, the presented result is still competitive enough to satisfy a desirable criterion of 10^5 ^RS cycles for emerging memory application^17^.

The effect of active device area on the RS characteristics was investigated with a set of the samples patterned into 6 different sizes as shown in Fig. 2(C). The resistance of the HRS rapidly increased up to 65% more as the active area decreased by 35 times, whereas the LRS showed relatively insensitive dependency on the various active cell sizes. The size-independent characteristic of the LRS indicates that the electrical conduction paths must be locally confined within the TaO_x_ matrix regardless of the cell size. The localized nature of the LRS will be correlated with its microscopic observation later.

The temperature dependency of the LRS and the HRS is shown in Fig. 2(d). In the LRS, the resistance level of the device rose up as the measurement temperature increased, which represents typical characteristic of current conduction in metals. On the other hand, the resistance of the HRS responded in the opposite way to the LRS. It indicates that the conduction paths formed in the LRS have metal-like characteristic and these metal-like conduction paths might weaken or diminish through the RESET operation. One possible model for this phenomenon is that the metal-like conductive phase was formed during the SET operation, similarly reported as oxygen deficient phase, Magnéli (Ti_n_O_2n-1_), in the TiO_x_ case^12^,^18^. The other is that metallic clusters are generated and connected with each another to finally build up the conduction paths between the top and the bottom electrodes. The conduction paths of the metallic clusters are likely to arise in the TaO_x_ based RS system because the thermodynamic equilibrium of the Ta-O system shows only two stable phases, oxide Ta_2_O_5_ and metal Ta, below 300 °C^7^,^15^,^19^,^20^. Moreover, two thermodynamic equilibrium phases (Ta_2_O_5_ and Ta) are not reactive with each other and the formed Ta phase can reside after the RS operation, resulting in the satisfactory LRS retention characteristic^7^,^15^. Although there are several metastable phases in Ta-O system, these oxide phases can only contribute to the circumstantial portion of the electrical conduction due to their insulating nature. Additionally, the electron conduction through those metastable phases has positive temperature dependency oppose to the metallic conduction.

**Figure 3:**
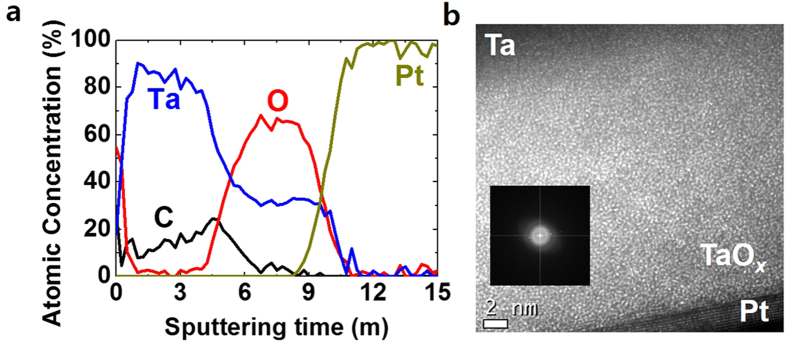
(**a**) The analyses of Auger electron spectroscopy (AES) and (**b**) the cross-sectional image and the related fast Fourier transformation (FFT) images of pristine Ta/TaO_x_/Pt device using high-resolution transmission electron microscope (HR-TEM)

To microscopically investigate the TaO_x_ thin film, HR-TEM and AES analyses were performed on the samples. Figure 3(a) shows the AES depth profile of the pristine Ta/TaO_x_/Pt device. The relative atomic concentration of Ta and O was estimated to be approximately 3:7 within the TaO_x_ layer. In addition, the top layer of Ta has negligible oxygen concentration to remain as a metal electrode. Figure 3(b) shows vertical structure of the Ta/TaO_x_/Pt device in the pristine state examined by HR-TEM. The inset of Fig. 3(b) provides the fast Fourier transformation (FFT) image of a smeared ring pattern in the TaO_x_ region, which indicates that the whole TaO_x_ thin film is composed of amorphous oxide phase homogeneously. The formation of the amorphous TaO_x_ layer is reasonable because the TaO_x_ thin film deposited at room temperature had insufficient activation energy to form any crystalline structure. The TaO_x_ thin film has the electrical property of an insulator in the pristine state as shown in Fig. 1(a) due to the homogeneously dispersed oxygen atoms.

**Figure 4:**
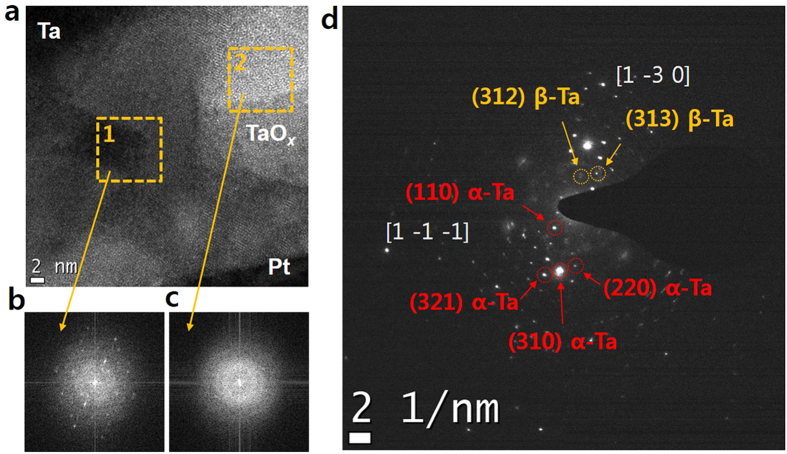
(**a**) The cross-sectional image of Ta/TaO_x_/Pt device after SET operation. (**b**) and (**c**) show the FFT images of dashed-yellow-square region marked ‘1’ and ‘2’, respectively. (**d**) the observed electron diffraction patterns (DP) of the Ta/TaO_x_/Pt device after the SET operation

In contrast to the homogeneous amorphous TaO_x_ thin film in the pristine state, some crystalline structures were observed after the SET operation as shown in Fig. 4(a). The most noticeable change is highlighted and marked as the dashed-yellow-square region ‘1’ in Fig. 4(a), and the related FFT image shown in Fig. 4(b). The electron diffraction pattern (DP) appearing as the spot pattern was observed in Fig. 4(b), which was not observed in the pristine state of the TaO_x_ thin film. It should be noted that the amorphous phase was still observed as shown in the dashed-yellow-square region ‘2’ in Fig. 4(a) and the related FFT image presented in Fig. 4(c). It suggests that the observed local crystalline structure should be formed during the SET operation.

To figure out the crystalline structures, the electron DP shown in Fig. 4(d) was analysed and identified as Ta-metal phases and its equi-directional crystallographic planes. The representative DPs of the Ta-metal phases were marked with dashed yellow and red circles in Fig. 4(d). There are two well-known phases of the Ta-metal in crystallographic point of view^21^,^22^. One is called ‘alpha-Ta’ (α-Ta), a body-centered cubic structure with lattice parameters of a = b = c = 3.305 Å, and the other is ‘beta-Ta’ (β-Ta), a primitive tetragonal structure with lattice parameters of a = b = 10.19 Å and c = 5.313 Å^21^,^22^. The representative planes of the alpha-Ta were found in the electron DP of the tested device in the LRS and indexed as (110) and (321) planes. In Fig. 4(d), the red and yellow dashed circles are related to the alpha-Ta and the beta-Ta, respectively. Based on the measured values from the electron DP image and the crystallographic equations described below, the extracted lattice parameters of (110) and (321) of the alpha-Ta planes were 3.29 Å and 3.35 Å, respectively.


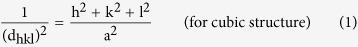



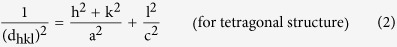



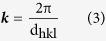


Where d_hkl_ is inter-planar spacing in real space, (h k l) is Miller indices, and ***k***is reciprocal vector when the Bragg condition is met. The lattice parameters extracted from each plane of the alpha-Ta have lattice strain less than 1% compared to the reference value. It seems comprehensible when the crystalline Ta-metal phases are surrounded by the amorphous TaO_x_. Moreover, the lattice mismatch can readily occur at the interfaces between the oxides and the metal phases. It is known that the lattice strain of less than 3% in the Ta metal phases is bearable to maintain their own crystalline structures even in thin film morphology^23^. Equivalent planes of (321) were also discovered to revalidate the crystalline structure as the alpha-Ta phase. Those planes undisputedly appeared at integer times of the reciprocal vector of (321) as supposed, simultaneously satisfying the symmetry of (321). It reassured that the lattice parameters obtained from the electron DP of the tested device in the LRS agree with ones of the alpha-Ta phase.

Similarly, the characteristic planes of the tetragonal beta-Ta were found to be identified as (312) and (313). The tetragonal structure of the beta-Ta requires more than two diffraction planes in order to extract each lattice parameters as shown in Eq. (2). The lattice parameters from those planes were a = b = 10.44 Å and c = 5.34 Å. Discrepancy between the reference and the extracted was respectively about 2% and 0.5% for ‘a’ and ‘c’, which is still laid in the elastic deformation range. Furthermore, equivalent planes of (312) of the beta-Ta phase were also observed and the symmetry of those planes was confirmed to reassure the crystalline structure as the beta-Ta phase. Although the beta-Ta phase known as a metastable phase is easily converted to the alpha-Ta above 750 °C, the beta-Ta phase can coexist with the alpha-Ta even in the thin film^24^. Thus the distinctive electron DP from two different metal-Ta phases illustrates that the alpha- and the beta-Ta phases coexist as a mixture within the TaO_x_ matrix after the SET operation and these metal phases should mainly contribute to the electron conduction in the LRS.

## Discussion

**Figure 5:**
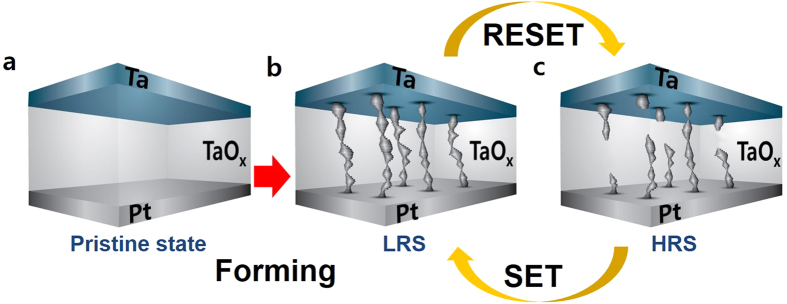
The schematic figures for a sequential RS cycle. (**a**), (**b**), and (**c**) show the pristine, the LRS, and the HRS of the Ta/TaO_x_/Pt device, respectively.

A series of the RS cycle is presented in Fig. 5 schematically. The RS cycle is initiated from the pristine state by forming process (Fig. 5(a)). The connection of the Ta metal phases is formed and the resistance of the device lowers to the LRS (Fig. 5(b)). Then the device transits to the HRS (Fig. 5(c)) through the RESET operation. The initial resistance of the pristine device, however, could not be fully recovered at the HRS as shown in Fig. 1(a) and the HRS showed the positive temperature dependency as an insulator or a semiconductor behaves. This indicates that some part of the Ta metal phase connection may disappear by recombination with oxygen during the RESET and the electrical conduction path may be shut conclusively. Further microscopic analysis on the device at the HRS is missing in this paper, which can explicitly confirm the Ta metal phases may be partially left after the RESET. Multiple trials were made on HR-TEM observation with the LRS TaO_x_ film during in-situ RESET process to paradoxically disclose the origin of the conduction path in the TaO_x_ film. Unfortunately, the RESET with the LRS samples prepared for the HR-TEM was extremely difficult and all the attempts ended up with failure. The conduction path in the TaO_x_ matrix has the restricted oxygen interchange border only in the both sides because the HR-TEM samples were sliced into a form of thin flake and the HR-TEM chamber was kept at ultra-high vacuum^12^. Under these circumstances, the in-situ observation of the RESET process was impossible to work out as desired.

In conclusion, the stable and promising RS performance of the Ta/TaO_x_/Pt device was demonstrated in this research. From the experimental results, the main factor of the LRS in the TaO_x_ based RS system turned out to be the Ta metal phases. The LRS of the tested Ta/TaO_x_/Pt device showed a typical temperature dependency characteristic of typical metals and device size independent nature. Moreover, thermodynamic equilibrium suggests that the only metallic phase in the Ta-O system is Ta metal. In addition, the electron DP of the device at the LRS indicated that two types of the Ta metal phases should exist in the TaO_x_ matrix. It was the first direct observation on the origin of the conduction path formed in the TaO_x_ film during the SET operation.

## Methods

A TaO_x_ thin film of approximately 30 nm thick was deposited on Pt/Ti/SiO_2_/Si substrates by reactive rf-sputtering technique at room temperature. During the TaO_x_ thin film deposition, the rf-sputtering power and the deposition pressure were maintained at 100 W and 2 mTorr, respectively. A top electrode of 30 nm thick Ta layer was deposited on the TaO_x_ film by dc-sputtering method at room temperature and patterned through photolithography and lift-off process, in order to measure the electrical properties of the TaO_x_ thin film. The current-voltage (*I*–*V*) characteristics of the fabricated samples were examined using a Keithley4200 semiconductor characterization system at room temperature. The pulse endurance measurement was also performed using a HP81110A arbitrary function wave generator. The electrical pulses of 100 nsec/2 V and 500 nsec/−2.5 V were applied sequentially for writing and erasing operation of the device. The material composition of the fabricated device was analysed by Auger electron spectroscopy (AES) using a scanning Auger nano-probe (ULVAC PHI-700). The sample surface was sputtered off for 30 sec prior to the AES analysis to eliminate native oxides and the undesired on the top surface. During the AES analysis, the sputtering rate was maintained at 15 nm/min estimated from SiO_2_ case. The vertical structure of the Ta/TaO_x_/Pt device was characterized by HR-TEM (The FEI Titan^™^ 80–300, 200 kV, a lateral resolution of less than 0.1 nm). The cross-sectional sample for the HR-TEM analysis was prepared by focused ion beam (FIB) technique. Firstly, the sample was milled into an approximately 30 μm thick slice by mechanical polishing. Secondly, the polished sample was bonded onto a one-sided Mo grid and fabricated to thinner area of about 30 nm thick by FIB (Analytical) technique.

## Additional Information

**How to cite this article**: Yang, M.K. *et al.* Direct evidence on Ta-Metal Phases Igniting Resistive Switching in TaO_x_ Thin Film. *Sci. Rep.* **5**, 14053; doi: 10.1038/srep14053 (2015).
